# Exploring the effects of physical exercise on inferiority feeling in children and adolescents with disabilities: a test of chain mediated effects of self-depletion and self-efficacy

**DOI:** 10.3389/fpsyg.2023.1212371

**Published:** 2023-09-15

**Authors:** Tongnian Yang, Hui Xiao, Xiaoyan Fan, Wenping Zeng

**Affiliations:** ^1^School of Physical Education and Health, Jiangxi Science and Technology Normal University, Jiangxi, China; ^2^Nanchang Qiyin School (School for the Deaf and Dumb), Jiangxi, China

**Keywords:** physical exercise, children and adolescents with disabilities, inferiority feeling, self-depletion, self-efficacy

## Abstract

**Objective:**

The purpose of this study was to investigate the effects of physical exercise on inferiority feeling of children and adolescents with disabilities and its mechanism of action, as well as the mediating role of self depletion and self-efficacy.

**Methods:**

The following scales were administered to 546 children and adolescents with disabilities (mean age 15.6 years): The Feelings of Inadequacy Scale, (FIS), the Self-Regulation Fatigue Scale (S-RFS), the General Self-Efficacy Scale (GSES), and the Physical Exercise Rating Scale.

**Results:**

(1) Physical exercise can directly and negatively predict inferiority feeling, self-depletion, and can directly and positively predict self-efficacy; self-depletion can directly and negatively predict self-efficacy. Similarly, self-depletion positively predicts inferiority feeling; physical exercise and self-efficacy can also directly and negatively predict inferiority feeling. (2) The indirect effect of the path with self-depletion as the mediating variable was – 0.05, the indirect effect of the path with self-efficacy as the mediating variable was – 0.09, and the indirect effect of the path with self-depletion and self-efficacy as the mediating variables was – 0.04. (3) The sum of all indirect effects was – 0.18, and the three indirect effects accounted for 15.6%, 28.1%, and 12.5% of the total effect, with mediating effect was 56.2%.

**Conclusion:**

Physical exercise can indirectly predict inferiority feeling in children and adolescents with disabilities through the independent mediation of self-depletion and self-efficacy, as well as through the chain mediation of both. This study supports that moderate physical exercise has a positive effect on the mental health of children and adolescents with disabilities, and that reducing self-depletion and improving self-efficacy are important ways to prevent inferiority feeling among children and adolescents with disabilities. It reveals the relationship between physical exercise and inferiority feeling and its mechanism of action, and further improves the research on the effect of physical exercise on inferiority feeling of children and adolescents with disabilities.

## Introduction

In 2023 UNICEF estimates that there will be 240 million children with disabilities aged 18 years and under (UNICEF, 2023). China has the largest population of children and adolescents with disabilities. According to the China Disability Survey, there are 3.87 million disabled people under the age of 14 in China, and there are approximately 2.46 million disabled children and youth between the ages of 6 and 14 (Wang et al., 2016). Studies have shown that there are differences in the psychological developmental stages of children and adolescents with disabilities (Kapsal et al., 2019). Compared to children and adolescents without disabilities, they may feel inferior due to some of their impairments, and due to this inferiority complex, they are not good at and afraid of human interaction (Jóhannsdóttir et al., 2022). Children and adolescents with disabilities are 3–4 times more likely to suffer from mental health problems than children and adolescents without disabilities (Einfeld et al., 2011). Physical exercise has a dual effect of strengthening physical fitness and physical and mental well-being, allowing the exerciser to experience the pleasures of exercise and to increase self-confidence, which can help to reduce inferiority feeling (Hale et al., 2023). Studies have shown that regular participation in physical exercise helps to improve and maintain the physical function and mental health of people with disabilities (Arbour-Nicitopoulos et al., 2018). The current low level of physical exercise participation among children and adolescents with disabilities in China necessitates effective physical exercise programes (Liu et al., 2019). Most studies on the effects of physical exercise on inferiority feeling have focused on normal people, but few studies have focused on children and adolescents with disabilities, and the question of “why physical exercise can reduce the level of inferiority feeling in children and adolescents with disabilities” has not been addressed (Chen et al., 2023). Based on this, this research study aims to explore the relationship between physical exercise on the inferiority feeling of adolescents with disabilities and the mediating role of self-depletion and self-efficacy to provide theoretical support for subsequent interventions.

## Theoretical background and formulation of research hypotheses

### Theoretical background

#### Self-depletion theory

Self-depletion is similar to the process of depleting mental energy during self-activation, i.e., a state of impaired executive functioning that results from the depletion of mental energy (Baumeister et al., 2018). According to the theory of self depletion proposed by Baumeister et al. (1998), individuals who are faced with potentially failing situations choose behaviors such as avoidance for the purpose of self-preservation. Self-depletion, in turn, can lead to negative experiences such as low motivation, pessimism and depression and inferiority feeling in individuals (Chen et al., 2011). Children and adolescents with disabilities may choose to avoid facing failures and setbacks during physical exercise by avoiding avoidance, a behavior that not only decreases their level of active participation in physical exercise, but also increases their doubts about their abilities and the development of inferiority feeling (Gaskin et al., 2010). Research in physical exercise science has shown that depletion of self-control is followed by a decrease in self-efficacy and affects exercise performance, and that self-depletion and self-efficacy are negatively correlated (Graham and Bray, 2015).

#### Self-efficacy theory

Self-efficacy theory was developed by Bandura (1997), and it is one of the most powerful theories for promoting behavior change (especially in the field of sports). Self-efficacy theory suggests that if a person has a high sense of self-efficacy for a particular activity, the likelihood that they will begin or continue to engage in that activity increases (Holm, 2018). In higher intensity physical activities, self-efficacy helps to shape an individual’s effort, emotional experience, and enjoyment (Samson and Solmon, 2011). Individuals with high self-efficacy attribute failure to the existence of a connection between things that can be changed and persevere in physical activity despite unfavorable circumstances, while low self-efficacy attributes failure to things that he cannot change, prompting them to give up exercise (Bandura, 1997). Individuals with weak self-efficacy, on the other hand, doubt their ability to deal with, and control potential threats from the environment, and thus experience strong stress states and anxiety arousal, and respond passively to the environment with a variety of protective withdrawal or defensive behaviors (Makara-Studziska et al., 2021). Children and adolescents with disabilities who have a high sense of self-efficacy will show more calmness and self-confidence when encountering difficulties and setbacks (Chong and Kua, 2016); and at the same time, the process of accomplishing challenges and resistance will enhance their self-efficacy, which in turn will prevent and reduce the emergence of inferiority feeling.

### Formulation of research hypotheses

#### The relationship between physical exercise and inferiority feeling

Inferiority feeling refers to the complex emotions of shame, shyness, trepidation, and even discouragement that result from an individual’s inferiority feeling (Li et al., 2023). Under the influence of inferiority feeling, children and adolescents with disabilities may excessively devalue themselves, and may even develop discrimination or even disgust towards themselves, eventually forming the extreme phenomenon of self-abandonment (Bai and Li, 2017); physical exercise has a two-way regulation effect on the psychology of children and adolescents with disabilities, which not only produces good psychological benefits but also improves poor psychology and enhances overall mental health benefits (Yang and Xiao, 2022). It was found that there was an interaction between the effects of physical exercise intensity on inferiority feeling, with students with disabilities in the small and medium exercise groups experiencing significantly lower levels of personality inferiority than those in the large exercise group (Qi, 2014). Medium-intensity physical exercise can strengthen the physical fitness of the exercisers and improve their motor skills, allowing them to feel pleasure, affirm their abilities and increase their confidence as they continue to improve their physical health and exercise levels, thus alleviating and reducing their inferiority feeling (Qin and Yuan, 2019); group exercise programs are better than individual exercise programs (Chen et al., 2016). Physical exercise can stimulate children’s innermost hopes and aspirations for a better future and help to enhance their confidence in life and eliminate inferiority feeling and autism (Cui and Pan, 2013). Therefore, hypothesis H1: physical activity is negatively associated with inferiority feeling among children and adolescents with disabilities.

#### The mediating role of self-depletion

Self-depletion is the temporary reduction in the ability or willingness to control oneself (including controlling the environment, self-control, making choices, and initiating actions) as a result of prior use of self-control (Ma et al., 2020). According to the self-control resource model, the psychological resources consumed by self-control are domain-specific, meaning that all resources are kept in a restricted resource pool and each self-control behavior requires self-control resources to perform the task, at which point the individual becomes ego-depleted (Pesce et al., 2021). It has been found that individuals experiencing self-depletion experience cognitive dissonance, which manifests as an underestimation of their own abilities, a negative evaluation of their ability to control the external environment, and more pessimistic expectations of the future (Inzlicht and Friese, 2019), resulting in feelings of inferiority. Regular physical exercise can enhance self-control and reduce the consumption of psychological resources required to complete the task of self-depletion (Xiong and Min, 2021). Physical exercise can enhance the body’s tolerance, reduce the self-loss from fatigue, and improve its self-control ability (Chan et al., 2013). The findings of Yiming et al. (2023) suggest that physical exercise can reduce people’s level of ego depletion and help reduce their negative effects such as inferiority feeling and depression. Thus, ego depletion may be an important bridge for physical exercise to influence inferiority feeling in children and adolescents with disabilities. For this reason, hypothesis H2 was proposed: Self-depletion mediates the relationship between physical exercise and feelings of inferiority feeling was proposed.

#### The mediating role of self-efficacy

Self-efficacy refers to an individual’s confidence and belief in their ability to perform a particular task (Wilde and Hsu, 2019). A decline in self-efficacy decreases the individual’s ability to adapt to the environment, and the individual is more likely to develop and display poor psychological behavioral tendencies in the face of stress (Wong et al., 2023); people with low self-efficacy have higher levels of inferiority feeling, more depressive disorders, and more negative coping styles when they encounter difficulties (Liu, 2022). Research has found that emotional self-efficacy is very closely related to negative emotions and that an individual’s ability to manage their negative emotions helps to eliminate them (Caprara et al., 2020). Negative emotions in inferiority feeling are caused by a low self-evaluation, and self-efficacy can influence an individual’s self-evaluation, hence the close relationship between self-efficacy and inferiority feeling. Lamberson and Wester (2018) found that self-efficacy was significantly negatively related to inferiority feeling after an investigation of self-efficacy and inferiority feeling, suggesting that the stronger an individual’s general self-efficacy, the higher the individual’s self-evaluation and This suggests that the stronger the individual’s general self-efficacy, the higher the individual’s self-evaluation and the lower the sense of inferiority. A large number of empirical studies have found that physical exercise is closely related to self-efficacy and that active participation in regular physical exercise helps to enhance individuals’ sense of efficacy in physical exercise, which in turn promotes general self-efficacy (Wu et al., 2022). Physical exercise is a good predictor of self-efficacy, and the higher the amount of exercise, the stronger the sense of self-efficacy (Yuan and Zhang, 2015). Physical exercise can directly influence the level of mental health, and also indirectly through self-efficacy (Jiang et al., 2017), and self-efficacy plays a partially mediating role in the effect of physical exercise on mental health (Sheng et al., 2016). Based on this, hypothesis H3: Self-efficacy mediates the relationship between physical exercise and feelings of inferiority feeling was proposed.

#### The chain mediating role of self-depletion and self-efficacy

Self-depletion is an important factor that influences self-efficacy (DeBono and Muraven, 2013). The greater the individual’s self-control and the more self-control resources required, the lower the self-depletion and the higher the self-efficacy produced by the individual (Swann et al., 2021). The self-control power model states that self-depletion negatively affects individuals and that as individual self-attrition behaviors occur, the self-control resources available to individuals continue to decrease and individuals are no longer able to use the only self-control resources available to support their self-efficacy, therefore, we can conclude that a decrease in self-efficacy can have a direct impact on the quality of self-control behaviors, and in severe cases can result in self-control failure (Yu et al., 2013). It has been shown that individuals with high willpower beliefs can resist the ego-depletion effect compared to low willpower beliefs and therefore do not negatively affect subsequent self-control tasks (Francis and Job, 2018). In summary, children and adolescents with disabilities who participate in physical exercise up to a high level of physical ability have better academic life skills, lower self-depletion, high self-efficacy, and lower levels of low inferiority feeling. Accordingly, hypothesis H4: Self-depletion and self-efficacy play a chain mediating role between physical exercise and inferiority feeling.

**Figure 1:**
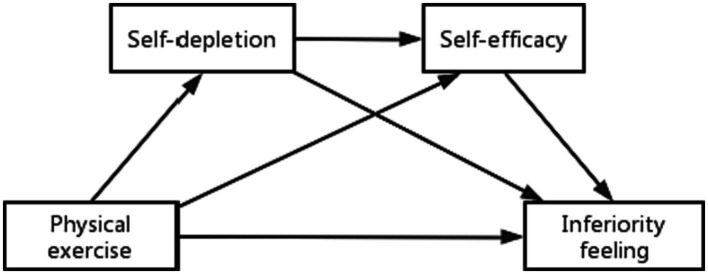
Chain intermediary model.

Based on this, this study intends to construct a chain mediation model (Figure 1), aiming to explore the influence of physical exercise on the inferiority complex of children and adolescents with disabilities and to reveal the mediating role of self-loss and self-efficacy, so as to provide theoretical references for the formation of mental health of children and adolescents with disabilities and appropriate physical exercise for persons with disabilities.

## Materials and methods

### Procedure and participants

The inclusion criteria for survey respondents are as follows: (1) Age between 10 and 18 years old. (2) the study subjects were diagnosed with hearing disability, speech disability and multiple disabilities by qualified professionals granted by China Health. (3) Students who were in the process of receiving junior and senior high school education. (4) Children with disabilities with the most basic athletic ability. In order to ensure the authenticity of the sample, the following exclusion criteria were set: the type of disability did not involve intellectual disability, cerebral palsy, Down’s syndrome, etc.

The study was conducted in strict accordance with the Declaration of Helsinki, and the anonymous survey involved was approved by the Ethics Committee of Jiangxi Science and Technology Normal University. With the approval of the teaching authorities of each school and the informed consent of the students and their parents, the survey was administered to the group by the classroom during the study period, using both paper and electronic questionnaires. In order to ensure the privacy and autonomy of the participants, we explained the purpose and significance of the study to the participants, and all the information collected in the questionnaires would be kept strictly confidential. Subsequently, the informed consent form and the questionnaire were distributed to the participants, and the researcher or classroom teacher explained the content of the questionnaire and the requirements for completing the questionnaire before the distribution of the paper questionnaire and the electronic questionnaire. The class teacher and the researcher were responsible for answering any questions during the questionnaire completion process. Subjects accepted the survey on a voluntary basis and answered anonymously. If any psychological discomfort occurs during the completion process, the survey can be terminated at any time. It was collected on the spot after completion.

This study used stratified whole-group sampling to survey 600 students in six grades from junior to senior high school in four schools in Nanchang and Ganzhou, Jiangxi. 100 students were selected from each grade and 600 questionnaires were distributed, 54 invalid questionnaires were excluded and 546 questionnaires were finally returned, with a valid return rate of 91%. Among them, 298 were boys, 248 were girls, 80 were junior high school students of grade 7 (14.6%), 74 were junior high school students of grade 8 (13.5%), 61 were junior high school students of grade 9 (11.2%), 120 were senior high school students of grade 10 (22.0%), 116 were senior high school students of grade 11 (21.3%) and 95 were senior high school students of grade 12 (17.4%); 238 were hearing disabled (43.6%), 168 were speech disabled (30.8%), and 140 (25.6%) with multiple disabilities; The age of the subjects ranged from 13 to 18 years, The average age is 15.6 years (SD = 0.87) (Table 1).

**Table 1:** The demographics of the participants.

Characteristics	Mean (SD)	*n*	%
The average age of young people with disabilities	15.6 (0.87)	546	
**Gender**			
Male		298	54.6
Female		248	45.4
**Grade level**			
Grade 7		80	14.6
Grade 8		74	13.5
Grade 9		61	11.2
Grade10		120	22.0
Grade11		116	21.3
Grade12		95	17.4
**Type of disability**			
Hearing impairment		238	43.6
Speech impediment		168	30.8
Multiple obstacles		140	25.6

### Measures and instruments

#### The feelings of inadequacy scale

The questionnaire was revised by Fleming and Courtney (1984), and the reliability of the Chinese version was tested by Cai et al. (2006). The questionnaire consists of 36 items (4 reverse scoring questions, 3, 6, 25 and 31 are reverse scoring questions). The questionnaire uses Likert-7 points to score, with a minimum score of 36 points and a maximum score of 252 points, involving 5 dimensions: self-esteem, appearance, physical ability, social confidence and academic ability. The Cronbach’s alpha coefficient for this questionnaire in this study was 0.864. The construction validity of the CFA model was good (*χ^2^*/df = 2.58, RMSEA = 0.04, NFI = 0.919, CFI = 0.950, SRMR = 0.06).

#### Self-regulation fatigue scale

The questionnaire was developed by Nes et al. (2013) and the Chinese version was revised by scholar Wang et al. (2015) with good reliability and validity. The questionnaire has 3 dimensions: cognitive, emotional, and behavioral, with 16 entries (6 reverse scoring questions). The questionnaire was scored on a Likert-5 point scale from ‘strongly disagree’ (1) to ‘strongly agree’ (5). The higher the score, the greater the degree of self-regulation fatigue, that is, chronic self-depletion, of which 1, 2, 5, 9, 14 are reverse scoring questions, with a score range of 16–80 points. The Cronbach’s alpha coefficient for this scale in this study was 0.823. The construct validity of the CFA model was good (*χ*^2^/df = 3.902, RMSEA = 0.064, NFI = 0.949, CFI = 0.973, SRMR = 0.026).

#### General self-efficacy scale

The questionnaire was translated and revised in Chinese by the scholar Wang et al. (2001) it has good reliability and validity and unidimensionality. The questionnaire has a total of 10 items, using Likert-4 point scoring, from ‘completely inconsistent’ (1) to ‘completely consistent’ (4), with a score range of 10–40 points. The higher the total score, the higher the self-efficacy of the participant. The Cronbach’s alpha coefficient for this scale in this study was 0.837. The construct validity of the CFA model was good (*χ*^2^/df = 2.07, RMSEA = 0.06, NFI = 0.93, CFI = 0.91, SRMR = 0.05).

#### Physical exercise rating scale

The Chinese version of the questionnaire was revised by scholar Liang (1994) with good reliability and validity. The scale is divided into 3 dimensions of physical exercise intensity, physical exercise time, and physical exercise frequency, with a total of 3 entries. The scale is scored on a Likert-5 point scale, corresponding to scores from 1 to 5, respectively. The formula for calculating the score of physical exercise volume = intensity × (time − 1) x frequency, with the lowest score being 0 and the highest being 100. Based on the scoring criteria, the subjects were categorized into 3 exercise groups: low, medium and high, where a score of ≤19 was categorized as a low exercise group, 20–42 as a medium exercise group, and ≥ 43 as a high exercise group. The Cronbach’s alpha for this scale in this study was 0.815. The construct validity of the CFA model was good (*χ*^2^/df = 4.109, RMSEA = 0.06, NFI = 0.939, CFI = 0.995, SRMR = 0.03).

### Data analysis

Descriptive statistics, correlation analysis, independent samples t-test and one-way ANOVA were performed on the variables of physical activity, low self-esteem, self-loss and self-efficacy using SPSS 26.0 statistical software, and the internal consistency coefficients and internal consistency reliabilities were measured using Cronbach’s α-value method.

This study used anonymous completion and reverse scoring of a small number of questions to control for common method bias. The data collected were tested for common method bias using Harman’s one-way validation method (Zhou and Long, 2004). The results of the principal component factor analysis without rotation showed that 21 factors with a characteristic root bigger than one were extracted and that the first factor explained 22.27% of the variance, which is less than the 40% criterion recommended by Hair et al. (1998), thus indicating that there was no common method bias in the data of this study.

Amos 26.0 was used to test for mediating effects. Main tests: goodness-of-fit test; direct effect relationship between physical exercise and inferiority feeling; mediating effect of self-depletion and self-efficacy; chain mediating effect of self-depletion and self-efficacy.

## Results

### Descriptive statistics and correlations

The Pearson correlation analysis of the four variables in this study is shown in Table 2. The results of the study showed that physical exercise was significantly negatively correlated with self-depletion (*r* = −0.174, *p* < 0.01), physical exercise was significantly negatively correlated with feelings of inferiority feeling (*r* = −0.211, *p* < 0.01), and physical exercise was significantly positively correlated with feelings of self-efficacy (*r* = 0.202, *p* < 0.01); self-depletion was significantly negatively correlated with feelings of self-efficacy (*r* = −0.433, *p* < 0.01), self-depletion was significantly positively correlated with inferiority feeling (*r* = 0.357, *p* < 0.01); self-efficacy was significantly negatively correlated with inferiority feeling (*r* = −0.181, *p* < 0.01), and the research hypothesis H1 was verified. The correlation of physical exercise, self-depletion, self-efficacy, and inferiority feeling were all significant, and this result also predicts a possible mediating role of self-weariness and self-efficacy between physical exercise and inferiority feeling, and is a prerequisite for the subsequent test of the mediating effect.

**Table 2:** Pearson correlation analysis for each variable.

Variables	M	SD	1	2	3	4
Physical exercise	21.78	9.52	1.00			
Self-depletion	46.68	10.77	−0.174^**^	1.00		
Self-efficacy	21.42	6.61	0.202^**^	−0.433^**^	1.00	
Inferiority feeling	119.01	23.69	−0.211^**^	0.357^**^	−0.181^**^	1.00

^**^Correlation is significant at the 0.01 level (2-tailed); *N* = 546.

The level of correlation between physical exercise, self-depletion, self-efficacy and inferiority feeling of children and adolescents with disabilities of different genders is shown in Table 3. Males had higher levels of physical exercise than females and also had higher levels of self-efficacy than females. However, females had a higher level of self-depletion than males and a higher level of inferiority feeling than males.

**Table 3:** Difference in gender.

	Gender	Number	M ± SD	*t*
Physical exercise	Male	298	24.32 ± 10.79	7.71^***^
	Female	248	18.73 ± 5.89	
Self-depletion	Male	298	44.21 ± 8.03	−6.39^***^
	Female	248	49.65 ± 11.26	
Self-efficacy	Male	298	23.15 ± 8.37	6.52^***^
	Female	248	19.34 ± 5.19	
Inferiority feeling	Male	298	112.43 ± 11.94	−8.23^***^
	Female	248	126.92 ± 25.62	

^***^Correlation is significant at the 0.001 level (2-tailed); *N* = 546.

### Differential analysis of physical exercise on dimensions of inferiority feeling among children and adolescents with disabilities

In order to observe more clearly and accurately the changes in the influence of the amount of physical exercise on the inferiority feeling of children and adolescents with disabilities, the subjects were divided into three groups according to the amount of exercise: high, medium and low, and the means of five dependent variables in the inferiority feeling were analyzed and examined by the different amounts of exercise. The results showed that the symptoms of inferiority feeling of children and adolescents with disabilities decreased with the increase of exercise volume, and the scores of self-esteem, social confidence, and physical Ability in the high exercise volume group were lower than those of the other two groups (see Table 4). The results of the test showed that the differences in the effects of different amounts of exercise on self-esteem, social confidence, and physical ability of children and adolescents with disabilities were statistically significant (*p* < 0.001), while the differences in the effects of different amounts of exercise on the academic ability and appearance of children and adolescents with disabilities were not statistically significant (*p* > 0.05). There was a statistically significant correlation between physical exercise and inferiority feeling, which provided solid evidence for the test of mediating effect.

**Table 4:** One-way analysis of variance on the effect of different amounts of exercise on inferiority feeling in children and adolescents with disabilities.

Variable	Low exercise group (*n* = 181)	Intermediate exercise group (*n* = 203)	High exercise group (*n* = 162)	*F*	*p*
Self-Esteem	31.35 ± 7.51	26.65 ± 4.18	19.51 ± 5.28	38.35	<0.001
Social Confidence	43.28 ± 11.39	36.47 ± 7.54	20.58 ± 6.71	76.16	<0.001
Academic Ability	24.92 ± 5.30	24.26 ± 4.37	21.36 ± 4.46	2.48	0.088
Appearance	18.66 ± 4.63	17.41 ± 3.82	17.64 ± 4.41	0.44	0.648
Physical Ability	21.53 ± 5.31	16.05 ± 4.22	14.47 ± 5.47	23.25	<0.001

### Chain mediation analysis

The study used AMOS 26.0 to develop structural equation modeling to analyze the chain mediating role of self-depletion and self-efficacy between physical exercise and feelings of inferiority feeling. Firstly, the modeling was based on the measurement of the observables physical exercise, self-depletion, self-efficacy, and feelings of inferiority feeling. It was found that each of the observables and the corresponding latent variables reached a significant level, confirming the validity of the instruments selected for this study. Secondly, the model was tested according to the mediation effect process, and the fitness of fit test of the mediation model was conducted using AMOS 26.0, which showed that: *χ*^2^/df = 4.246, RMSEA = 0.07, GFI = 0.947, NFI = 0.939, RFI = 0.918, IFI = 0.951, TLI = 0.934, and CFI = 0.951. The model met the fit criteria for each indicator, with a good fit (see Table 5).

**Table 5:** Model fit.

Model indicators	*χ^2^*/df	RMSEA	GFI	NFI	RFI	IFI	TLI	CFI
Coefficient	4.246	0.075	0.947	0.939	0.918	0.951	0.934	0.951
Standard	<5.00	<0.08	>0.90	>0.90	>0.90	>0.90	>0.90	>0.90

**Figure 2:**
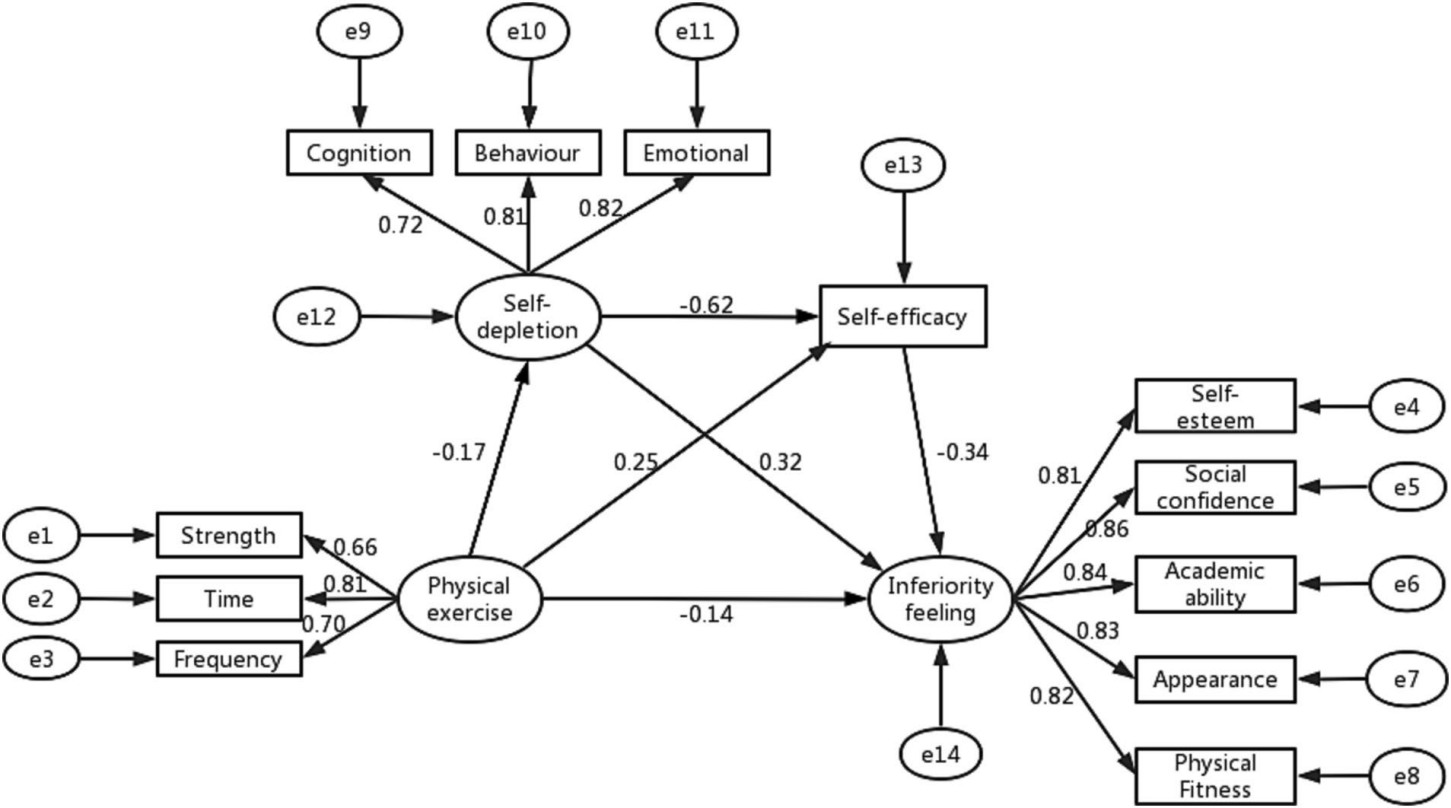
Chain mediated effect model.

In order to verify the mediating role of self-depletion and self-efficacy, the significance of the specific mediating effect was estimated using the bias-corrected nonparametric percentile Bootstrap method, setting the Bootstrap sampling number to 5,000, and calculating the mediating effect as well as the 95% confidence intervals. According to the results of mediation effect analysis in Table 6 and Figure 2, it can be seen that the indirect effect of self-depletion as a mediating variable was – 0.05 (95% CI = [−0.068, −0.032]), self-efficacy as a mediating variable was – 0.09 (95% CI = [−0.114, −0.066]), and self-depletion and self-efficacy as a mediating variable was – 0.04 (95% CI = [−0.056, −0.024]), and the total indirect effect was – 0.18 (95% CI = [−0.219, −0.142]). The 95% confidence intervals of the mediating effects of the three paths mentioned above did not contain 0, indicating that the mediating effects were significant, and the three paths of the indirect effect accounted for 15.6%, 28.1%, and 12.5% of the total effect. The results of the study confirmed hypotheses H2, H3, and H4. Therefore, the chain mediating role of self-depletion and self-efficacy in the effect of physical exercise on feelings of inferiority feeling was established.

**Table 6:** Analysis of model mediation effects.

Pathways of influence	Standardized mediated effect values	Effectiveness ratio	95% Confidence interval	
			LLCI	ULCI
Physical exercise → inferiority feeling (test hypothesis 1)	−0.14	43.8%	−0.177	−0.103
Physical exercise → self-depletion→ Inferiority feeling (test hypothesis 2)	−0.05	15.6%	−0.068	−0.032
Physical exercise → self-efficacy → Inferiority feeling (test hypothesis 3)	−0.09	28.1%	−0.114	−0.066
Physical exercise → self-depletion → Self-efficacy → Inferiority feeling (test hypothesis 4)	−0.04	12.5%	−0.056	−0.024
Total indirect utility	−0.18	56.2%	−0.219	−0.142

## Discussion

### The relationship between physical exercise and inferiority feeling

The results of the present study showed differences in physical exercise and inferiority feeling among children and adolescents with disabilities in terms of differences by gender, a result that is consistent with previous studies (Monzani et al., 2008; Queralt et al., 2016). Research has shown that boys typically experience higher levels of physical activity when using more play areas and equipment (Anthamatten et al., 2014), while girls typically show higher levels of enjoyment when walking, creative tasks, sitting, hiding, and relaxing (Hyndman and Chancellor, 2015). Female adolescents with physical disabilities are at particular risk if they do not conform to the current image of beauty presented in the media, and they tend to feel that the social environment in which they live is negative (Retznik et al., 2017). Girls with physical disabilities have weaker self-esteem and more pronounced feelings of inferiority than boys, and they perceive their disability as more burdensome than boys (Monzani et al., 2008).

The present study showed a significant negative correlation between physical exercise and inferiority feeling of children and adolescents with disabilities, which verified hypothesis H1 and was consistent with the results of previous studies (Liu, 2010; Qin and Yuan, 2019). Exercise can lead to a series of physiological changes, which have an impact on mental health. Studies have shown that exercise can increase the release of 5-hydroxytryptamine (Bruschetta, 2020). Increased neuronal expression factors such as brain-derived neurotrophic factor (Jamali et al., 2020), increased β-endorphin release (Wipfli et al., 2011), and stimulation of the inferior colliculus-pituitary–adrenal axis (Shwayhat et al., 2014). These changes can directly change the emotional state or activate other pathways such as hippocampal nerves. These changes may be the physiological mechanism of exercise affecting inferiority feeling. It has been suggested that the mechanism by which exercise promotes mental health may be that physical exercise reduces symptoms of inferiority feeling, depression, and anxiety, improves quality of life, and mitigates cognitive decline by providing social support, enhancing the exercise experience, and bringing about a range of physiological changes (Li et al., 2015). The effect of physical exercise on mental health has been widely confirmed, and the items of physical exercise, time, intensity and frequency are the key indicators that affect the inferiority feeling, which are less involved. Previous studies have shown that a group exercise program of 45–50 min of moderate intensity physical exercise three times a week can effectively reduce students’ inferiority feeling, promote the level of physical self-esteem and psychological capital, and improve psychological health (Chen et al., 2016; Qin and Yuan, 2019). Physical exercise has different effects on the health of different types of disabled children and adolescents. Studies have found that children and adolescents with physical disabilities who participate in physical activity have improved their athletic ability and skills, goal control, social skills, peer interaction, and self-confidence (Arbour-Nicitopoulos et al., 2018); after 3 months of skating training, parents found that children with hearing impairment had improved in self-esteem and behavior (Dursun et al., 2015); children with muscular dystrophy participating in physical activities, such as swimming, can develop social skills and increase self-confidence and fun (Valle et al., 2016).

This study focused on the relationship between physical exercise and inferiority feeling, and found that children and adolescents with disabilities who participated in medium and high exercise volume had lower levels of inferiority feeling, which is consistent with the results of the previous study, and further supports the idea that long-term regular medium-and high-intensity aerobic exercise is more positive and beneficial to the development of inferiority feeling. Children and adolescents with disabilities should pay attention to the intensity, time and frequency of exercise, and achieve medium and high exercise can have a more significant effect on the inferiority feeling of children and adolescents with disabilities.

### The mediating role of self-depletion

The results of this study show that self-depletion plays a mediating role between physical exercise and inferiority feeling of disabled children and adolescents, which verifies Hypothesis H2. First, the present study found that self-depletion significantly and positively predicted inferiority feelings, a result that fits the self-depletion (Tan et al., 2012), which states that an individual’s resources for self-control are limited at a given time, and that insufficient resources for self-control lead to nonadaptive behaviors and mental health problems. Due to their impairments, children and adolescents with disabilities suffer from ridicule and psychological damage in early childhood, resulting in barriers to communication with normal children and adolescents, which can induce their negative emotions, and these controlling behaviors cause self-depletion (Zhang, 2012). When individuals are in a state of self-depletion, it can cause psychological depletion, which in turn increases the likelihood that individuals will develop a variety of externalizing (for example, aggression and addictive behaviors) and internalizing (for example, anxiety, depression, and inferiority feelings) problems (Yu et al., 2013). High self-depletion compared to low self-depletion results in cognitive dissonance as well as inferiority feeling, which manifests as positive illusions about one’s abilities and personality, a lower sense of mastery, and a decrease in one’s control over the external environment (Forestier et al., 2022). The results of related studies also point out that low self-control leads to the development of inferiority feeling, which means that the depletion of self-control resources is one of the most important reasons for the emergence of inferiority feelingin individuals (Iranmanesh et al., 2021).

Adolescents are a critical period of growth for individuals, with immature thinking and low self-control, while facing academic pressure, leading to depletion of self-control resources, however, through regular physical exercise, the self-depletion caused by academic pressure can be restored (Wang and Mao, 2015). Different physical exercise modalities produce different benefits for mental health, and the effect of physical exercise on the psyche has a bidirectional regulatory function, which not only produces good psychological balance benefits, but also improves people’s bad psychology and enhances emotional overall mental health benefits (Yang and Xiao, 2022). The more athletic children and adolescents with disabilities are, the more adequate their stored self-control resources are, the lower the degree of self-depletion is, and thus the internalization of socially expected standards can be accomplished more smoothly, with a relatively low inferiority feeling, and ultimately, the psychological aspects will move towards a benign development.

### The mediating role of self-efficacy

The results of the present study showed that self-efficacy mediates the relationship between physical exercise and inferiority feeling among children and adolescents with disabilities, validating Hypothesis H3, which is consistent with the results of previous studies (Sheng et al., 2016; Jiang et al., 2017). Research in the trans-theoretical model of behavior change has suggested that child and adolescent self-efficacy increases sequentially with progression through the stages of exercise behavior change, showing significant stage change characteristics (Lu et al., 2022). The middle school to high school stage is a time when individuals’ general self-efficacy tends to gradually increase as their thinking gradually matures and develops and they become more capable of recognizing things (Wang and Zhao, 2011). Changes in the self-efficacy of people with disabilities correlate with changes in their level of physical exercise, and self-efficacy is a very important factor that influences the participation of people with disabilities in physical exercise (Motl et al., 2013). Physical exercise enhances individual willpower and self-efficacy by improving physical fitness and causing positive physical and psychological changes when individuals participate in physical exercise (Yang, 2013). Exercise interventions, including low-to moderate-intensity aerobic exercise, may be most effective in improving students’ mental health (Herbert, 2022). At the same time, a high sense of self-efficacy enables individuals to cope with the stress of internal and external environments more brighter and confident, while increasing self-efficacy can effectively mitigate the negative effects of inferiority feeling. Therefore, physical exercise can improve the self-efficacy of children and adolescents with disabilities, thus indirectly reducing the level of inferiority feeling.

Self-efficacy, as a useful variable in regulating the mental health of children and adolescents with disabilities, not only improves the persistence and durability of physical exercise for children and adolescents with disabilities, but also enhances positive emotional experiences. On this basis, we should stimulate the motivation of children and adolescents with disabilities to exercise, formulate detailed exercise programs for special groups, and improve their self-efficacy and willingness to participate in physical exercise.

### The chain mediating role of self-depletion and self-efficacy

The results of this study indicate that self-depletion has a significant negative predictive effect on self-efficacy, which is consistent with previous findings (Graham et al., 2017), and that physical exercise can have an effect on inferiority feeling in adolescents with disabilities through a chain mediating effect of self-depletion and self-efficacy, testing hypothesis H4. The behaviors that motivate children and adolescents with disabilities to take the initiative to participate in physical exercise are intrinsic psychological needs and extrinsic social support, and the internal and external cycles increase the sense of autonomy of children and adolescents with disabilities to participate in physical exercise and their positive emotional experience (Wu et al., 2017). The more psychological satisfaction they get from participating in physical exercise, the more self-control they have, and the lower the corresponding self-attrition (Sun and Yu, 2014). High self-depletion prompts perceptions of protection of psychological resources, which undermines self-control and reduces self-efficacy (Chow et al., 2015). Low self-depletion compared to high self-loss, low self-depletion allows for the use of self-control resources more effectively, responds more effectively to immoral events (Yam et al., 2014), increases willingness to help others (Jin et al., 2021), increase pro-social behavior (Joosten et al., 2015), individuals are more effective in increasing propensity to exercise and increasing exercise behavior due to reduced stress (Schöndube et al., 2017), more effective in addressing inferiority feeling associated with psychological problems and thus improve self-efficacy (Huang and Yang, 2015). Children and adolescents with disabilities who have a high level of physical activity experience fun and pleasure in exercise, and the long-term pressure due to low self-esteem is released, which improves the executive control function of the brain, reduces self-depletion, and self-efficacy brings about a stronger ability to repair the psyche, and indirectly reduces the level of inferiority feeling.

### Limitations and future prospects

This study explored the relationship between physical exercise and inferiority feeling and its intrinsic mechanism, and constructed a chain mediation model of self-depletion and self-efficacy between physical exercise and inferiority feeling, which is of theoretical and practical value to the development of mental health of children and adolescents with disabilities. At the same time, it provides ideas for exercise intervention and prevention of inferiority feeling in children and adolescents with disabilities. However, this study has some limitations. First, this study used psychometrics, which is a cross-sectional study, and may have some limitations in predicting the relationship between variables. Longitudinal studies can be used in future research to reveal the mechanism of physical exercise’s effect on inferiority feeling. Exercise can also be used to intervene in the sense of inferiority feeling among children and adolescents with disabilities, and the study can be implemented in terms of the type and level of physical exercise. Secondly, this study used a combination of paper questionnaires and questionnaire star measurements, which is a self-reported form of survey with a large number of questions that may affect the results of the study. Future research could collect data from sources such as parents of students with disabilities and educators to reduce measurement error and further explain the relationship between variables. Finally, this study focused on children and adolescents with disabilities who have hearing impairments, speech impairments, and multiple impairments; other disability groups were not addressed, and future research should include other disability groups in the study. Research results show that physical exercise not only improves physical health but also promotes mental health, and special schools should create a quality exercise environment for students with disabilities and cultivate their interest in exercise. At the same time, schools should develop reasonable exercise programs according to the age of children and adolescents with disabilities, the type of disability, physical ability, relevant policies and other factors. In the process of improving the inferiority feeling of children and adolescents with disabilities, more attention should be paid to the psychological benefits brought by physical exercise, which not only reduces self-depletion, but also improves self-efficacy, which are two important ways to reduce the inferiority feeling. In addition, it was found that parent–child relationship in the family as well as family sports are crucial to the healthy development of children and adolescents with disabilities. Therefore, in the process of improving the inferiority feeling of children and adolescents with disabilities in the future, it can be realized by strengthening the parent–child relationship as well as family sports activities.

## Conclusion

Physical exercise directly predicts inferiority feeling levels in children and adolescents with disabilities. Self-depletion and self-efficacy play an important mediating role between physical exercise and self-efficacy. There were 3 mediating pathways, namely the separate mediating effect of self-depletion, the separate mediating effect of self-efficacy, and the chain mediating effect of self-depletion and self-efficacy. The chain mediating effect model constructed in the study has some reference value for the prevention and alleviation of inferiority feeling among children and adolescents with disabilities, and in the future, it is recommended that families, schools, and the state pay more attention to the physical exercise of children and adolescents with disabilities and actively carry out physical education; reduce the level of ego depletion and enhance self-efficacy, and then alleviate the inferiority feeling of children and adolescents with disabilities.

## Data availability statement

The raw data supporting the conclusions of this article will be made available by the authors, without undue reservation.

## Ethics statement

The studies involving humans were approved by Jiangxi Science and Technology Normal University. The studies were conducted in accordance with the local legislation and institutional requirements. Written informed consent for participation in this study was provided by the participants’ legal guardians/next of kin. Written informed consent was obtained from the individual(s), and minor(s)’ legal guardian/next of kin, for the publication of any potentially identifiable images or data included in this article.

## Author contributions

TY designed the study and wrote the manuscript. HX and XF collected and analyzed the data. TY and HX revised the manuscript. WZ collected and analyzed the data and revised the manuscript. All authors contributed to the article and approved the submitted version.

## Funding

This study was supported by the Research Project on Teaching Reform of Higher Education Institutions in Jiangxi Province (Project No. JXJG-22-10-2), the Provincial Project of Basic Education in Jiangxi Province (Project No. SZUJKTY2022-1098), and the Research Project on Humanities and Social Sciences of Colleges and Universities in Jiangxi Province (Project No. TY18114).

## Conflict of interest

The authors declare that the research was conducted in the absence of any commercial or financial relationships that could be construed as a potential conflict of interest.

## Publisher’s note

All claims expressed in this article are solely those of the authors and do not necessarily represent those of their affiliated organizations, or those of the publisher, the editors and the reviewers. Any product that may be evaluated in this article, or claim that may be made by its manufacturer, is not guaranteed or endorsed by the publisher.
